# Impact of COVID-19 on financial returns: a spatial dynamic panel data model with random effects

**DOI:** 10.1007/s43071-022-00025-8

**Published:** 2022-09-01

**Authors:** Anna Gloria Billé, Massimiliano Caporin

**Affiliations:** 1 Department of Statistical Sciences, Alma Mater Studiorum University of Bologna, Bologna, Italy; 2 Department of Statistical Sciences, University of Padua, Padua, Italy

**Keywords:** Covid-19, Financial crisis, Spatio-temporal modeling, C23, C33, G01, G15, C58

## Abstract

Using a dataset including financial market returns and volatility proxies for several countries, we analyzed the impact of Covid-19 deaths on the financial economy. From the modeling perspective, we consider a spatial panel data model for returns and a spatial dynamic panel data for volatilities. Proper marginal effects are calculated to exploit information on short- and long-term effects. A Chow test is used to identify the existence of a structural break in each series. Our empirical evidence shows that in the first weeks of the Covid-19 outbreak, until mid-March 2020, the identified break date, the spatial effect of Covid-19 deaths was statistically significant, leading to a contraction in returns and an increase in risk. Moreover, the effects disappeared in the remaining months as the financial markets moved back to pre-crisis levels, causing a decrease in the overall risk. Our evidence supports the behavioral impact of the pandemic on financial markets.

## Introduction

The diffusion of the Covid-19 pandemic has had an extremely relevant impact on societies, with consequences at different levels; for example, social interaction, learning approaches, and economic activities. Governments have made several efforts to prevent virus diffusion and have supported the pharmaceutical industry in identifying a vaccine in the shortest possible time. We are now close to a possible exit from the crisis, thanks to the availability of several vaccines, but the limits on their production and diffusion still leave areas of the world exposed to contagion. Furthermore, the real impact of the pandemic has not yet been fully evaluated, and uncertainty remains as to how quickly we will escape the crisis induced by Covid-19.

Financial markets responded to the spread of the pandemic with a clear drop in the level of the stock indexes, which decreased even more than 40% compared to the value of the indexes at the beginning of 2020.^1^ Later, the financial markets began to recover, a process that was completed, in many cases, at the end of 2020.^2^ However, uncertainty surrounding the impact of the crisis on the real economy, and the fact that the recovery of the financial markets was supported by few economic sectors, put a challenge on the future development of the markets.

In this paper, we focus the attention on the financial market’s movements during the pandemic outbreak and in the following months. Our interest lies in the evaluation of the impact of Covid-19 diffusion among different countries around the world, taking into account several controls. In particular, we are interested in determining whether the increase in Covid-19-related deaths in a country affects the evolution of the financial markets of the country itself and neighboring countries, both at the contemporaneous level and with a delay of one week.^3^ This can be done by specifying a spatial panel data model for the variable of interest, either the returns or the volatility proxy, where we include a spatial dependence on the deaths recorded in neighboring countries. Such effects, if present, will be intelligibly driven by behavioral aspects rather than by economic fundamentals. This is in line with the recent contribution of Mamaysky (2020), which detected a hypersensitivity of financial markets to news in the first weeks of Covid-19 diffusion. The increase in deaths in neighboring countries might be perceived by the financial market of a given country as an indication of further future increases in deaths in the country. The presence of such an effect, even unrelated to the various containment decisions made by the countries, can find an explanation only in behavioral reactions to the pandemic diffusion. In particular, the reaction is evident when focusing on neighboring countries, while it is not related to deaths in the own country.

From a methodological point of view, our study is related to the literature that analyses economic and health data from a spatial perspective. In this field, we mention (Al-Awadhi et al. 2020; Ali et al. 2020; Ashraf 2020; Erdem 2020; Hafner 2020; Lyocsa and Monlar 2020; Zhang et al. 2020; Alexakis et al. 2021) that focus on financial market data and (Guliyev 2020) for Covid-19 data. The contribution of Alexakis et al. (2021) is closely related to ours in terms of both the focus on international financial markets and the use of spatial econometric tools. However, some differences can be found. While Alexakis et al. (2021) used the number of cases, we focus on deaths; it is known that the deaths dynamic lags the number of cases by about two weeks. However, our intuition is that the number of cases includes asymptomatic infections, which could provide a different view from those associated with deaths. In addition, the number of cases might be measured across countries, at the country level, with different statistical approaches and with different diagnostic tools, thus leading to quantities that would not be comparable and also characterized by different dispersion. Therefore, we believe that deaths could be a more precise measure of the impact of Covid-19 diffusion and the effects it has on a given country. Moreover, we controlled for the change in the market reaction by contrasting the first weeks of the virus’s diffusion with the remaining part of 2020, thus utilizing a much longer period compared to previous studies. We restrict our attention to 2020 without including the most recent months where the diffusion of the vaccine could introduce additional elements, such as heterogeneity in the vaccination campaign across countries.

The most relevant distinction of our work compared to that of Alexakis et al. (2021) is on model specification, which substantially differs: even if we both specify an SDPD model (Lee and Yu 2010; Parent and LeSage 2012; Elhorst et al. 2013; Shi and Lee 2017) with spatially lagged covariates, our research additionally considers time-lagged covariates as well as the inclusion of two control variables to account for potential asymmetries in stock returns, i.e. the Trade Weighted U.S. Dollar Index and the WTI Crude Oil Prices. Instead of considering individual fixed effects, we rely on the individual random effects, allowing for the inclusion of a correlation among them. Moreover, both serial and spatial autocorrelation among the error terms are included. Finally, the model specification also incorporates a dummy variable that interacts with the other regressors to identify the presence and impact of a potential structural break in the data. Notably, we accompany the model estimation with the evaluation of the marginal effects, distinguishing between the direct and indirect effects, and we also analyze them both at the short-term and long-term.

We show that the number of deaths in neighboring countries impacts the financial market evolution of a given country. This is novel evidence that complements recent findings in the behavioral financial literature; in particular, we refer to the contributions of Al-Awadhi et al. (2020), Baig et al. (2021), and Smales (2021). Furthermore, by taking advantage of a longer sample compared to early studies, we observe that the effect is present only in the first weeks of the Covid-19 outbreak and then disappears quickly, according to the evidence in the news reported by Mamaysky (2020); the result appears both when looking at the model estimates and when focusing on the marginal effects. However, the analysis of the latter shows evidence of heterogeneity across countries. Evidence on the role played by the Covid-19 pandemic only in the first weeks of its spread is present in both the returns and the evolution of the risk and supports the existence of a behavioral impact of the pandemic.

The paper proceeds as follows. Section 2 describes the data and identifies a break in the evolution of the series. Section 3 introduces the model we adopt to analyze the evolution of financial markets, while Sect. 4 focuses on empirical evidence.

## Data

The balanced panel dataset consists of weekly financial stock market indexes and the weekly number of deaths in 41 countries, i.e. Australia, Austria, Belgium, Brazil, Canada, Chile, China, Colombia, Denmark, Egypt, Finland, France, Germany, Hungary, India, Indonesia, Ireland, Israel, Italy, Japan, Malaysia, Mexico, Netherlands, New Zealand, Norway, Peru, Philippines, Poland, Portugal, Russia, Singapore, South Africa, South Korea, Spain, Sweden, Switzerland, Taiwan, Thailand, Turkey, UK, USA. We collect Covid-19 cases from December 24, 2019, to December 09, 2020, with a total number of observations equal to $N\times T = 41\times 51 = 2,091$. We download the equity data from the Thomson Reuters Eikon database, while the epidemic data have been collected from: https://opendata.ecdc.europa.eu/Covid-19/casedistribution/csv. In order to work with homogeneous market indexes, we download the MSCI country indexes, all expressed in the United States Dollar (USD); the availability of such indexes drives the selection of countries we made. In this way, the analyses we perform will evaluate the impact of the pandemic on the equity market combined with the effect of the currency. We note that, different from most of the articles dealing with the impact of Covd-19 on the equity markets, we chose to work with weekly data to avoid any effect due to asynchronous market trading activity within a day, due to the different reporting choices of Covid-19 deaths, and due to possible delays or asynchronicity in deaths recording. These elements will be smoothed and their impact reduced by working with a weekly time series. In addition, such a choice would allow one to give a proper value to the deaths recorded during the week-ends or during days where equity markets were closed (which adds another source of heterogeneity when working with daily data).

**Fig. 1:**
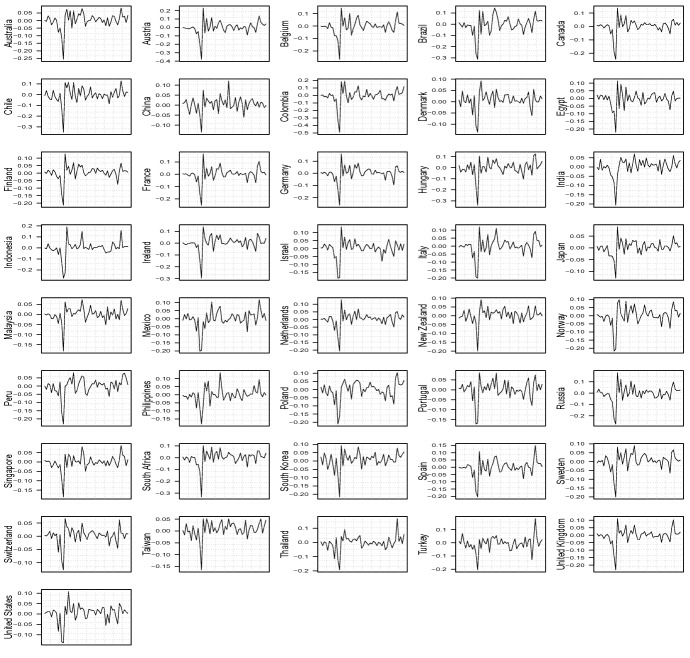
Time series of log–returns with weekly data

**Fig. 2:**
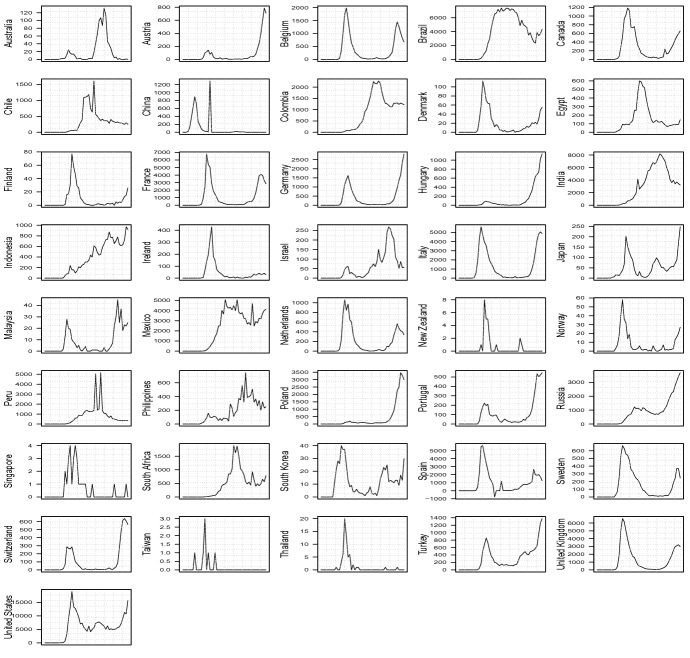
Time series of deaths with weekly data

Additional control variables refer to daily data of Trade Weighted U.S. Dollar Index to control for the possible role exerted by the exchange rate market, and WTI Crude Oil Prices available at the Federal Reserve Bank of St.Louis (FRED) website; we use them as global proxies of economic activity available at a weekly frequency; we decided to not include additional variables monitored at the US-market levels commonly adopted as global proxies as we believe that the will not be representative of the dataset we consider, characterized by a high level of heterogeneity. Figures 1 and 2 show the evolution of the series. In the former, the equity series by country are transformed into log–returns, see Sect. 3, whereas in the latter, the number of deaths is in level.

**Fig. 3:**
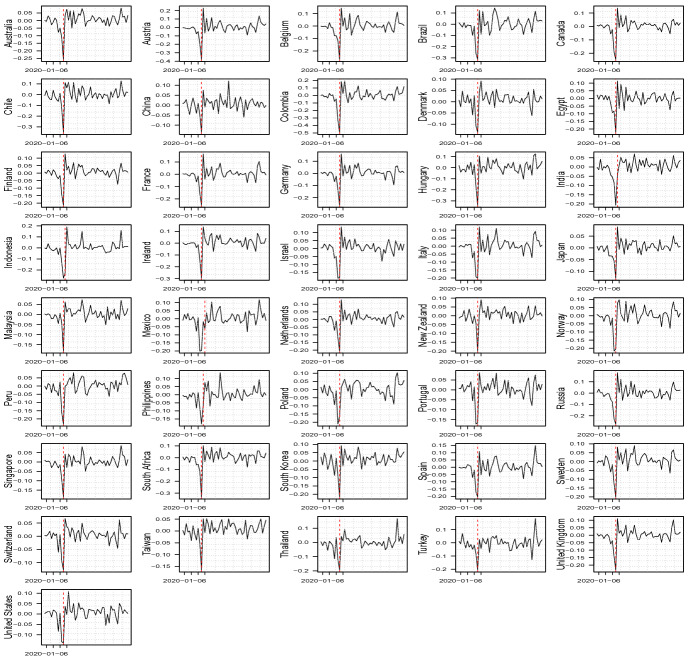
Change points identified through the Chow test for all the series in log–returns

### A chow test to identifying a potential structural break

We checked for the presence of at least one structural break in the weekly financial market series by considering a simple Chow test for each country. To identify the change point, we took the log–return series and separately regressed them on a constant term over the whole sample; see Fig.  3.

As we can observe, an important change point within the week in the middle of March 2020. We recall that we work with deaths, and thus the break date is identified two weeks after the start of the sensible increase in the number of cases, coherently with the empirical evidence on the diffusion of the pandemic. Therefore, we built a temporal dummy variable *D* with 0 assigned before the potential change point (11 weeks) and 1 after it (the remainder 38 weeks). This break identifies a possible change in the financial market reaction to the diffusion of Covid-19. In the first period, markets were clearly hit by the pandemic, with relevant drops in the index levels, as highlighted by the large negative movements in the returns, with a reaction driven, at least in part, by behavioral effects and possibly by limitations on trading (including the ban of short selling).

In the second period, the enforcement of prescriptions to reduce virus diffusion and the research for a vaccine acted in two ways. On the one hand, the behavioral effect tends to disappear as more information is released to the market, and at the same time, the knowledge of the disease improves. On the other hand, the impact on the economic sectors is heterogeneous, with some sectors negatively hit by the pandemic (e.g. the Retail and Travel and Leisure sectors), with others benefiting from the situation (in particular, the Healthcare and Technology sectors).

The joint effect of these two forces leads to markets retracing from the negative drop and then laterally moving while waiting for more clear information about the spread of the disease, the availability of the vaccine, and, more relevantly, with a better measurement of the long-term impact of the pandemic on the real economy. We also note that the break date we identify is equivalent to that detected by Mamaysky (2020) when analyzing the impact of news on the evolution of financial markets. In the next section, we introduce a model that we use to monitor the effect of Covid-19 on financial markets, driven by the diffusion of the disease in bordering countries, i.e., from a spatial perspective.

## Model specification

We specify a spatial–temporal model aimed at describing the impact of the number of deaths due to Covid-19 to financial markets returns. We denote by $I_{i,t}$ the equity market index of the country *i* on the last open market day of the week *t*. Then, the equity return is built as $r_{i,t} = \log (I_{i,t}) - \log (I_{i,t-1})$. Both the Trade Weighted U.S. Dollar Index ($TI_t$) and the Crude Oil Prices Index ($OPI_t$) are transformed into logarithmic returns in the same way. For Covid-19 deaths, we aggregate daily data within each calendar week, that is, from Monday to Sunday, and denote by $c_{i,t}$ (where the label "c" states for Covid-19) for country *i* and week *t*. In general, the panel we consider has dimensions $N=41$ and $T=51$, so we aggregated the sum of the observations over time to obtain weekly data and study weekly log–returns with $T = 50$. The number of deaths is then taken in time first differences ($\Delta$) to eliminate their trend components, so the number of weeks reduces to 49, considering the covariates at time $t-1$.^4^ Finally, a temporal dummy variable $d_t$ with 0 before the potential change point (as identified in Sect. 2.1), and 1 otherwise, is included in the model specification to account for a potential structural break in log–returns.

We exclude the possibility of including unobserved common factors modeled as interactive fixed effects in our specifications; see Shi and Lee (2017), Moon and Weidner (2015), Bai (2009). The reason is that we do not assume a global (strong) dependence that affects the financial markets returns of the selected countries. Coherently with the previous assumption, we do not include the contemporaneous spatio-temporal term ${\mathsf {W}}{\mathbf {y}}_t$, leaving to the control variables the role of controlling for lagged common factors, and letting the residuals capture the common contemporaneous dependence across financial markets. On the contrary, we include the spatio-temporal term ${\mathsf {W}}{\mathbf {y}}_{t-1}$ to describe financial market returns to capture the neighboring–country effects of returns with a delay of one week. Unlike common factors, this process captures delayed spatial interactions, also known as a global (weak) type of dependence. We do not include the dynamic term ${\mathbf {y}}_{t-1}$ since financial markets returns are in general unpredictable conditional on their own lagged values (serial dependence, if present, is weak and economically irrelevant). The introduction of the lagged spatio-temporal term would detect if some form of predictability induced by neighboring countries is present.

The structural spatio-temporal model is therefore specified as follows:

1 $$\begin{aligned} {\mathbf {y}}_{t}&= \gamma _1{\mathsf {W}}{\mathbf {y}}_{t-1} + \beta _1\Delta {\mathbf {x}}_{c,t}+\beta _2\Delta {\mathbf {x}}_{c,t-1}+ \delta _1{\mathsf {W}}\Delta {\mathbf {x}}_{c,t}\nonumber \\&\quad +\delta _2{\mathsf {W}}\Delta {\mathbf {x}}_{c,t-1} +\mu _1TI_{t-1} + \mu _2OPI_{t-1} + \nonumber \\&\quad + \mu _3|TI_{t-1}| + \mu _4|OPI_{t-1}| + \beta _0\iota _nd_t +\gamma _2{\mathsf {W}}{\mathbf {y}}_{t-1}\times \iota _nd_t \nonumber \\&\quad + \beta _3\Delta {\mathbf {x}}_{c,t}\times \iota _nd_t + \beta _4\Delta {\mathbf {x}}_{c,t-1}\times \iota _nd_t\nonumber \\&\quad +\delta _3{\mathsf {W}}\Delta {\mathbf {x}}_{c,t}\times \iota _nd_t + \nonumber \\&\quad + \delta _4{\mathsf {W}}\Delta {\mathbf {x}}_{c,t-1}\times \iota _nd_t +\mu _5TI_{t-1}\times \iota _nd_t \nonumber \\&\quad + \mu _6OPI_{t-1} \times \iota _nd_t +\mu _7|TI_{t-1}| \times \iota _nd_t \nonumber \\&\quad + \mu _8|OPI_{t-1}| \times \iota _nd_t + {\mathbf {u}}_{t} \end{aligned}$$

where ${\mathbf {y}}_{t}$ is an *n*–dimensional vector of financial log–returns in week *t*, that is, ${\mathbf {r}}_t$, $\gamma {\mathsf {W}}{\mathbf {y}}_{t-1}$ is the spatio–temporal lagged dependent variable with ${\mathsf {W}}$ a time–invariant weight matrix of known constants, ${\mathbf {x}}_{c,t}$ is an $n \times 1$ matrix of the exogenous number of deaths due to Covid-19, and $\iota _n$ is *n*–dimensional vector of ones. The model allows for both a contemporaneous and lagged impact of Covid-19 on financial markets returns. Moreover, we distinguish between the direct impact, coming from each country’s number of deaths, and the indirect impact, i.e., the effect coming from neighboring countries. To avoid potential endogeneity problems between log–returns and the economic indicators $TI_t$ and $OPI_t$, we included their lagged values in the model; endogeneity could be present, as financial market movements could influence economic indicators at the contemporaneous weekly frequency, and economic indicators could also influence financial market performances. Furthermore, the absolute values of both $TI_{t-1}$ and $OPI_{t-1}$ are taken into account to capture potential asymmetries. Inclusion of the dummy variable $d_t$ duplicates the previous components, allowing us to evaluate whether the first phase of Covid-19 diffusion plays a different role from the post-break period. Finally, ${\mathbf {u}}_{t}$ is the generic vector of error terms that includes the random effects $\varvec{\alpha }$ defined as $\varvec{\alpha }_i \sim {\mathcal {N}}\left( 0, \sigma ^2_\alpha \right)$ and $\varvec{\varepsilon }_{t} \sim {\mathcal {N}}\left( 0, \sigma ^2 I\right)$. Several specifications of the error terms are defined in the following according to the type of correlation included.

A different specification has been used to model a proxy of the weekly volatility, defined by resorting to the absolute value of weekly log–returns, i.e. $|{\mathbf {y}}_{t}|$. In this specification, we replace the spatial effect ${\mathsf {W}}{\mathbf {y}}_{t-1}$ by the time-lagged variable $|{\mathbf {y}}_{t-1}|$ and the spatio–temporal variable in absolute value ${\mathsf {W}}|{\mathbf {y}}_{t-1}|$. These two components will help capture both the serial dependence present in financial market volatility and the possible interdependence due to a spatial component. The structural equation is as follows:

2 $$\begin{aligned} |{\mathbf {y}}_{t}|&= \psi _1|{\mathbf {y}}_{t-1}| + \gamma _1{\mathsf {W}}|{\mathbf {y}}_{t-1}| + \beta _1\Delta {\mathbf {x}}_{c,t} + \beta _2\Delta {\mathbf {x}}_{c,t-1}\nonumber \\&\quad + \delta _1{\mathsf {W}}\Delta {\mathbf {x}}_{c,t} + \delta _2{\mathsf {W}}\Delta {\mathbf {x}}_{c,t-1} +\mu _1TI_{t-1} + \mu _2OPI_{t-1} + \nonumber \\&\quad + \mu _3|TI_{t-1}| + \mu _4|OPI_{t-1}| + \beta _0\iota _nd_t + \psi _2|{\mathbf {y}}_{t-1}|\times \iota _nd_t \nonumber \\&\quad + \gamma _2{\mathsf {W}}|{\mathbf {y}}_{t-1}|\times \iota _nd_t + \beta _3\Delta {\mathbf {x}}_{c,t}\times \iota _nd_t \nonumber \\&\quad + \beta _4\Delta {\mathbf {x}}_{c,t-1}\times \iota _nd_t + \nonumber \\&\quad + \delta _3{\mathsf {W}}\Delta {\mathbf {x}}_{c,t}\times \iota _nd_t + \delta _4{\mathsf {W}}\Delta {\mathbf {x}}_{c,t-1}\times \iota _nd_t \nonumber \\&\quad +\mu _5TI_{t-1}\times \iota _nd_t + \mu _6OPI_{t-1} \times \iota _nd_t + \mu _7|TI_{t-1}|\times \iota _nd_t + \nonumber \\&\quad + \mu _8|OPI_{t-1}| \times \iota _nd_t + {\mathbf {u}}_{t} \end{aligned}$$

where the autoregressive term $|{\mathbf {y}}_{t-1}|$ has been added.

Starting from model specifications in Eqs. (1) and (2), we consider several generalizations to deal with correlated/independent random effects, spatial and serial correlation in the error terms for both of them. In all the following specifications, we consider the inclusion of *serial correlation* to be necessary to describe financial returns and volatility, so we exclude cases without serial correlations in the idiosyncratic error terms. In the model specification in Eq. (1) the addition of serial correlation can capture any effect that cannot be captured by the reasonable exclusion of the dynamic term $y_{t-1}$, while in the model specification in Eq. (2) the addition of serial correlation can capture any remaining effect of the correlation. Random effects can be correlated or independent. Let ${\mathbf {u}}_t$ be the general vector of the error terms in both Eqs. (1) and (2). In the former case (Kapoor et al. 2007) the specification is as follows:

3 $$\begin{aligned} {\mathbf {u}}_t&= \phi {\mathsf {W}}{\mathbf {u}}_t + \varvec{\varepsilon }_{t} \nonumber \\ \varvec{\varepsilon }_{t}&= \varvec{\alpha }+ {\mathbf {v}}_t \end{aligned}$$

where ${\mathbf {v}}_t \sim {\mathcal {N}}\left( 0, \sigma ^2 I\right)$. A peculiarity of this specification is that the same spatial process is imposed through the reduced form model in both random effects $\varvec{\alpha }$ and idiosyncratic error terms ${\mathbf {v}}_t$. (Millo 2014) proposed a different specification with the addition of serial correlation in the residuals:

4 $$\begin{aligned} {\mathbf {u}}_t&= \varvec{\alpha }+ \varvec{\varepsilon }_{t} \nonumber \\ {\mathbf {u}}_t&= \phi {\mathsf {W}}{\mathbf {u}}_t + {\mathbf {v}}_t \nonumber \\ {\mathbf {v}}_t&= \zeta {\mathbf {v}}_{t-1} + {\mathbf {e}}_t \end{aligned}$$

where now the spatial process is directly imposed on both the random effects and the error terms, and ${\mathbf {e}}_t \sim {\mathcal {N}}\left( 0, \sigma ^2 I\right)$. The model with correlated random effects and serial correlation is labeled with *SEM2SRRE* (Spatial Error Model with Serial Correlation and Correlated Random Effects). A non-nested specification considers independent random effects with spatial autocorrelation in the idiosyncratic error terms and serial correlation in the remainder part of the error term (Baltagi et al. 2007) as

5 $$\begin{aligned} {\mathbf {u}}_t&= \varvec{\alpha }+ \varvec{\varepsilon }_{t} \nonumber \\ \varvec{\varepsilon }_{t}&= \lambda {\mathsf {W}}\varvec{\varepsilon }_{t} + {\mathbf {v}}_t \nonumber \\ {\mathbf {v}}_t&= \zeta {\mathbf {v}}_{t-1} + {\mathbf {e}}_t \end{aligned}$$

where again ${\mathbf {e}}_t \sim {\mathcal {N}}\left( 0, \sigma ^2 I\right)$. In this case, the model is labeled with *SEMSRRE* (Spatial Error Model with Serial Correlation and Independent Random Effects); see (Millo 2014). By setting $\varvec{\alpha }= 0$ in Eq. (5), we finally consider two sub-specifications without random effects, the former with both spatial and serial correlation labeled with *SEMSR* (Spatial Error Model with Serial Correlation) and the latter with only serial correlation, setting $\lambda = 0$, labeled with *SR* (Serial Correlation). The whole estimation procedure is performed using the function spreml in the package splm in R (Millo and Piras 2012).

We define the weight matrix ${\mathsf {W}}$ through a geographic definition of distance based on the *k*–nearest neighbour criterion among countries, with the number of neighbors set at $k=8$. Then we row–normalise such that $\sum _j w_{ij} = 1 \;\; \forall i$. We recognize that alternative definitions based on economic information can also be used, for instance, by using trades of goods or air flows among countries. However, we believe these aspects are appropriately proxied by geographical distance. This is also consistent with a behavioral interpretation of the Covid-19 deaths, which is perceived as more relevant when associated with closer countries, in a geographical sense.

Finally, in addition to model estimates, we calculate the short-term and long-term marginal impacts of the model in Eq. (1), see Debarsy et al. (2012) and Elhorst (2014, Sec. 4.6). As our interest is focused on Covid-19, the short-term and long-term marginal effects are calculated with respect to the variable $\Delta {\mathbf {x}}_{c,t}$ only. For the model in Eq. (1) in terms of log-returns, they are, respectively, defined as

6 $$\begin{aligned} \frac{\partial {\mathbb {E}}\left( {\mathbf {y}}_t\right) }{\partial \Delta {\mathbf {x}}_{c,t}}\mid _t&= \left[ \beta _1{\mathbf {I}}+ \delta _1{\mathsf {W}}+\beta _3\text{ diag }{\left( \iota _nd_t\right) } +\delta _3{\mathsf {W}}\text{ diag }{\left( \iota _nd_t\right) }\right] \nonumber \\ \frac{\partial {\mathbb {E}}\left( {\mathbf {y}}_t\right) }{\partial \Delta {\mathbf {x}}_{c,t}}&= \left[ {\mathbf {I}}- \gamma _1{\mathsf {W}}-\gamma _2{\mathsf {W}}\text{ diag }{\left( \iota _nd_t\right) }\right] ^{-1} \nonumber \\&\quad \times \left[ \left( \beta _1 + \beta _2\right) {\mathbf {I}}+ \left( \delta _1 +\delta _2\right) {\mathsf {W}}+ \left( \beta _3 + \beta _4\right) \text{ diag }{\left( \iota _nd_t\right) }\right. \nonumber \\&\left. \quad + \left( \delta _3 +\delta _4\right) {\mathsf {W}}\text{ diag }{\left( \iota _nd_t\right) }\right] , \end{aligned}$$

while for the model in Eq. (2), focusing on volatility, the same are defined as

7 $$\begin{aligned} \frac{\partial {\mathbb {E}}\left( {\mathbf {y}}_t\right) }{\partial \Delta {\mathbf {x}}_{c,t}}\mid _t&= \left[ \beta _1{\mathbf {I}}+ \delta _1{\mathsf {W}}+\beta _3\text{ diag }{\left( \iota _nd_t\right) } +\delta _3{\mathsf {W}}\text{ diag }{\left( \iota _nd_t\right) }\right] \nonumber \\ \frac{\partial {\mathbb {E}}\left( {\mathbf {y}}_t\right) }{\partial \Delta {\mathbf {x}}_{c,t}}&= \left[ (1-\psi _1){\mathbf {I}}-\psi _2\text{ diag }{\left( \iota _nd_t\right) } - \gamma _1{\mathsf {W}}-\gamma _2{\mathsf {W}}\text{ diag }{\left( \iota _nd_t\right) }\right] ^{-1} \nonumber \\&\quad \times \left[ \left( \beta _1 + \beta _2\right) {\mathbf {I}}+ \left( \delta _1 +\delta _2\right) {\mathsf {W}}+ \left( \beta _3 +\beta _4\right) \text{ diag }{\left( \iota _nd_t\right) }\right. \nonumber \\&\quad \left. + \left( \delta _3 +\delta _4\right) {\mathsf {W}}\text{ diag }{\left( \iota _nd_t\right) }\right] . \end{aligned}$$

The results of Eq. (6) (similarly for Eq. 7) are two matrices with dimension $n \times n$ which are also time-varying due to the presence of the dummy $d_t$ (i.e., the marginal short- and long-term effects change when $d_t$ moves from 0 to 1). The marginal effects are thus different before and after the break identified in the data. At a given point in time, the averages of the main diagonal elements reveal the *short* and *long-term direct effects*, respectively, while the averages of the off-diagonal elements of the two matrices show the *short* and *long-term indirect effects*, i.e. due to neighboring countries only. Cross-sectional unit-specific effects can also be shown as in Billé and Rogna (2021). This can reveal a sensible variation in both the *direct* and the *indirect* effects between countries. In addition to the marginal effects, we propose to calculate the standard deviations of the short- and long-term direct and indirect effects to identify the potential heterogeneity among the analyzed countries.

**Table 1:** Regressions’ Results with Weekly Data. Random Effects specifications: (1) spatial and serial errors with correlated random effects (SEM2SRRE), (2) spatial and serial errors with independent random effects (SEMSRRE), (3) spatial and serial errors (SEMSR) and (4) only serial correlation (SR)

	Log–returns				Volatility			
	SEM2SRRE	SEMSRRE	SEMSR	SR	SEM2SRRE	SEMSRRE	SEMSR	SR
$$|{\mathbf {y}}_{t-1}|$$	–	–	–	–	0.764$$***$$	0.763$$***$$	0.840$$***$$	0.866$$***$$
$${\mathsf {W}}{\mathbf {y}}_{t-1}$$	– 0.0642	– 0.695	– 0.588	0.629$$***$$	–	–	–	–
${\mathsf {W}}|{\mathbf {y}}_{t-1}|$	–	–	–	–	– 0.0741	– 0.0743	– 0.0878	0.166$\cdot$
${\mathbf {x}}_{c,t}$	– 0.000007	– 0.000007	– 0.000007	– 0.000003	– 0.000007	– 0.000007	– 0.000008	– 0.00002
${\mathbf {x}}_{c,t-1}$	0.00004$\cdot$	0.00004$\cdot$	0.00004$\cdot$	0.00003	– 0.00003$*$	– 0.00003$*$	−0.00003$*$	– 0.00002
$${\mathsf {W}}{\mathbf {x}}_{c,t}$$	– 0.0002$$*$$	– 0.0002$$*$$	– 0.0002$$*$$	– 0.0001	0.0001$$**$$	0.0001$$**$$	0.0001$$**$$	0.00009$$\cdot $$
${\mathsf {W}}{\mathbf {x}}_{c,t-1}$	0.000007	0.000007	0.000007	– 0.0001	– 0.00005	– 0.00005	– 0.00007	0.00003
$$OPI_{t-1}$$	– 0.169	– 0.168	– 0.171	– 0.283$$***$$	0.316$$*$$	0.313$$*$$	0.326$$*$$	0.313$$***$$
$$TI_{t-1}$$	3.33$$\cdot $$	3.31$$\cdot $$	3.35$$\cdot $$	5.84$$***$$	−4.86$$**$$	-4.85$$**$$	– 4.89$$**$$	– 5.23$$***$$
$$|OPI_{t-1}|$$	– 1.09$$***$$	– 1.09$$***$$	– 1.09$$***$$	– 0.947$$***$$	0.973$$***$$	0.968$$***$$	9.56E–01$$***$$	0.824$$***$$
$$|TI_{t-1}|$$	1.39	1.38	1.40	2.51$$***$$	– 1.11	– 1.26	– 1.35	– 2.16$$***$$
*D*	0.00163	0.00162	0.00162	0.00217	0.0195$$***$$	0.0189$$***$$	0.0176$$***$$	0.0180$$***$$
$$|{\mathbf {y}}_{t-1}|\times D$$	–	–	–	–	–0.422$$***$$	-0.4.22$$***$$	−0.429$$***$$	-0.462$$***$$
$${\mathsf {W}}{\mathbf {y}}_{t-1}\times D$$	0.128	0.134	0.121	– 0.665$$***$$	–	–	–	–
${\mathsf {W}}|{\mathbf {y}}_{t-1}|\times D$	–	–	–	–	0.0563	0.0565	0.0399	– 0.248$*$
${\mathbf {x}}_{c,t}\times D$	0.917	0.000009	0.000009	– 0.000001	0.000007	0.000008	0.000008	0.00001
${\mathbf {x}}_{c,t-1}\times D$	– 0.00004$\cdot$	– 0.00004$\cdot$	– 0.00004$\cdot$	– 0.00003	0.00003$*$	0.00003$*$	0.00003$*$	2.06E–05
$${\mathsf {W}}{\mathbf {x}}_{c,t}\times D$$	0.0002$$*$$	0.0002$$*$$	0.0002$$*$$	0.00014$$\cdot $$	−0.000148$$**$$	– 0.000149$$**$$	– 0.000149$$**$$	– 0.00009$$\cdot $$
${\mathsf {W}}{\mathbf {x}}_{c,t-1}\times D$	– 0.000008	– 0.000008	– 0.000007	0.000124	0.0000538	0.0000529	0.000077	– 0.0000377
$$OPI_{t-1}\times D$$	0.0997	0.0987	0.102	0.227$$***$$	– 0.382$$*$$	– 0.379$$*$$	−0.392$$**$$	– 0.367$$***$$
$$TI_{t-1}\times D$$	– 3.24$$\cdot $$	– 3.22$$\cdot $$	– 3.26$$\cdot $$	– 5.83$$***$$	4.58$$**$$	4.57$$**$$	4.57$$**$$	4.99$$***$$
$$|OPI_{t-1}|\times D$$	1.23$$***$$	1.23$$***$$	1.23$$***$$	1.07$$***$$	– 0.903$$***$$	-0.898$$***$$	– 0.889$$***$$	– 0.765$$***$$
$$|TI_{t-1}|\times D$$	– 1.44	– 1.43	– 1.45	– 2.54$$***$$	0.239	0.394	0.384	1.45$$**$$
$$\phi $$	0.000000007	0.00000002	–	–	0.0267$$**$$	0.0265$$**$$	–	–
$$\lambda $$	0.7330$$***$$	0.7342$$***$$	0.7318$$***$$	–	0.7608$$***$$	0.7615$$***$$	0.7558$$***$$	–
$$\zeta $$	– 0.0418$$\cdot $$	– 0.0422$$\cdot $$	– 0.0432$$\cdot $$	0.0282	– 0.2804$$***$$	– 0.2797$$***$$	– 0.3085$$***$$	– 0.3070$$***$$

Signif. codes: 0 ’***’ 0.001 ’**’ 0.01 ’*’ 0.05 ’.’ 0.1 ’ ’ 1

## Empirical evidence

In Table 1 we report the main regression results. First, focusing on the returns, the significance and impact of the coefficients seem to be similar in the specifications SEM2SRRE, SEMSRRE, and SEMSR, while some difference emerges for the model with only the spatial correlation in the residuals (SR). Indeed, SR reveals numerous significant coefficients due to the exclusion of the spatial autoregressive parameter in the error terms, that is, $\lambda$, for the regressions in terms of *log–returns*. Moreover, the serial correlation $\zeta$ lost its significance once the spatial autoregressive term $\lambda$ is excluded in the regression of *log–returns*. Both the lagged Trade Weighted U.S. Dollar Index ($TI_{t-1}$) and the Crude Oil Prices Index ($OPI_{t-1}$), as well as their absolute values, increase their impacts and significance when excluding $\lambda$ (SR case). However, the introduction of spatial correlation seems to be relevant in our dataset. We link this to the possibility that the spatial component in the error term proxies, at least partially, the contemporaneous correlation that exists between financial market returns (around 0.55, on average, in our data) and is not captured by other components in the model. Furthermore, the exclusion of spatial correlation in innovations leads to a statistically significant impact of the spatio–temporal coefficient, that is, ${\mathsf {W}}{\mathbf {y}}_{t-1}$, which is not consistent with the efficiency of the financial markets. Consequently, we consider the SEMSR specification to be the most appropriate for capturing the behavior of returns.

Moving to the volatility case, we note a stronger role for the lagged component, in line with the known serial dependence that characterizes the evolution of market risk. With regard to the error components, both the serial and spatial correlations are statistically significant, and, differently from the returns case, also fixed effect correlation seems to be present (the coefficient is statistically significant but with a limited magnitude). Therefore, to model volatility, we focus on the SEM2SRRE specification.

The most interesting evidence appears when considering the role of the Covid-19 variable, as well as the results before and after the break date. We start by noticing that the macroeconomic factors impact almost cancel out after the break date; this is happening both on the returns and on our volatility proxy. We might interpret this as a reaction of the market to information external to the macroeconomic fundamentals whose evolution is highly impacted by the pandemic’s diffusion. When focusing on the volatility, the positive (as expected) impact of the lagged volatility proxy almost halves after the break date. This indicates a decrease in the persistence of volatility and an increase in the role of information coming from markets.

Regarding the Covid-19 variables, a greater role is played by the contemporaneous spatially lagged variable, i.e., ${\mathsf {W}}{\mathbf {x}}_{c,t}$, before and after the structural break and both in terms of *log–returns* and *volatility*. As expected, the sign of the coefficient is opposite for returns and volatility: in the first, the impact during the first Covid-19 phase is negative, leading to a contraction of returns; in the second, the coefficient is positive, inducing an increase in volatility; this last result is consistent with the results in Baig et al. (2021). Consequently, the spread of the pandemic and the number of deaths in neighboring countries affect the risk and return of the country’s financial market. Interestingly, *own* deaths do not statistically affect returns or risk. This finding is consistent with the evidence in Smales (2021) for the US market, but opposite to that of Al-Awadhi et al. (2020) for China; this is in line with the possibility that a heterogeneous impact at the country level could be present, as opposed to the general trend highlighted by our panel model.

In the second phase, after the break we identified, the effect disappears. In fact, the coefficients are statistically significant but of opposite sign compared to the first part of the sample and of comparable magnitude. We read the different behavior as a consequence of several aspects: first of all, Covid-19 acts as an external shock to the financial markets, leading to a contraction of returns and an increase in volatility; second, after the diffusion of the pandemic at the global level, the impact of deaths in neighboring countries is losing impact as the pandemic becomes a sort of common factor with impacts across all countries and markets; while in the first period, behavioral effects could be present, they disappear when the pandemic is diffusing in all countries. Furthermore, our evidence is consistent with those of Mamaysky (2020), where the overreaction of the financial market to news extends up to mid-March 2020. In our case, the reaction is proxied by death cases in neighboring countries, leading to a sort of hypersensitivity of financial markets to this variable.

**Fig. 4:**
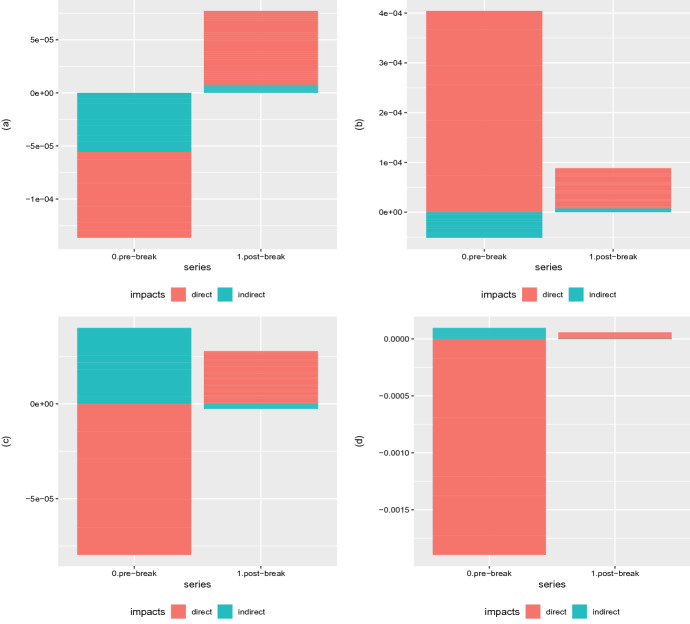
Short-term (on the left) and Long-term (on the right) Marginal Effects in terms of Log–Returns (**a**, **b**) and Volatility (**c**, **d**) with respect to the number of deaths variable ${\mathbf {x}}_{c,t}$ for models (**a**, **b**) SEMSR and (**c**, **d**) SEM2SRRE, respectively

**Fig. 5:**
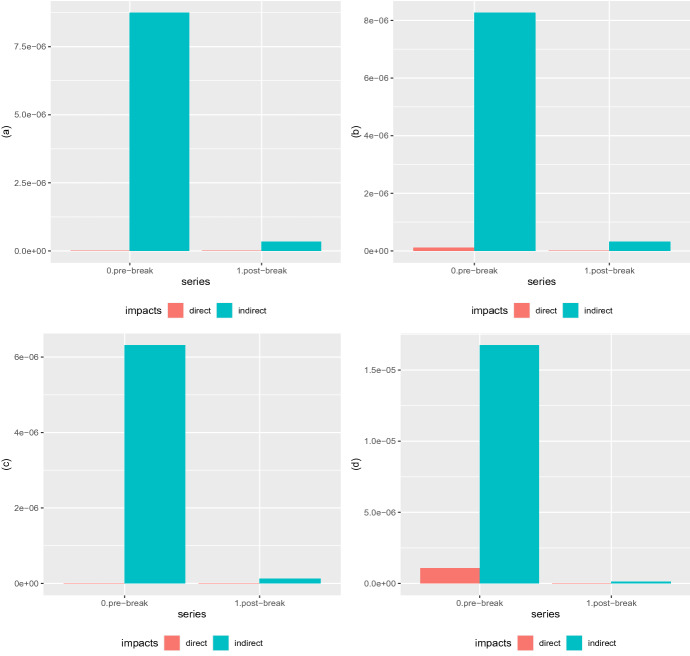
Standard deviations (SDs) of the short-term (on the left) and long-term (on the right) marginal effects in terms of log–returns (**a**, **b**) and Volatility (**c**, **d**) for models (**a**, **b**) SEMSR and (**c**, **d**) SEM2SRRE, respectively

Figure 4 shows the short- and long-term marginal effects in terms of logarithmic returns and volatility for the two reference specifications, SEMSR and SEM2SRRE, respectively.^5^ For the return case, the direct and indirect short-term effects are negative in the first weeks of Covid-19 diffusion, thus supporting the adverse effect of the pandemic on financial markets. In the same period, the long-term effects have an opposite sign, with the direct being positive and only the indirect one still on the negative side, in line with the negative effect due to the diffusion of the pandemic on neighboring countries. When looking at the post-break period, we do observe that both the short- and long-term marginal effects sensibly reduce and are dominated by a positive direct effect, suggesting that the Covid-19 impact is concentrated in the first period.

Moving to volatility, we have confirmation of the role played by the pandemic in the first period, while after the break the effect disappears. Unlike the return case, we note that the direct short-term effect is negative while the indirect effect is positive, leading to an increase in the risk in response to the deaths in neighboring countries. Long-term marginal effects have a similar structure, with a much larger role played by direct effects.

The standard deviations of direct and indirect effects for the short and long term, Fig. 5, show that in the first period the response is heterogeneous between countries, while in the post-break period the dispersion decreases.^6^ In particular, the heterogeneity is much higher for indirect effects, signaling that countries were characterized by very different reactions to deaths in neighboring countries.

## Conclusions

Using a dataset spanning the first and second Covid-19 waves, we observe an unexpected impact of the Covid-19 deaths of a country on the neighboring countries’ financial markets’ return and risk. This behavioral effect is present during the Covid-19 outbreak and disappears when additional information on the pandemic and vaccine is distributed.

## Figures and Tables

**Fig. 6:**
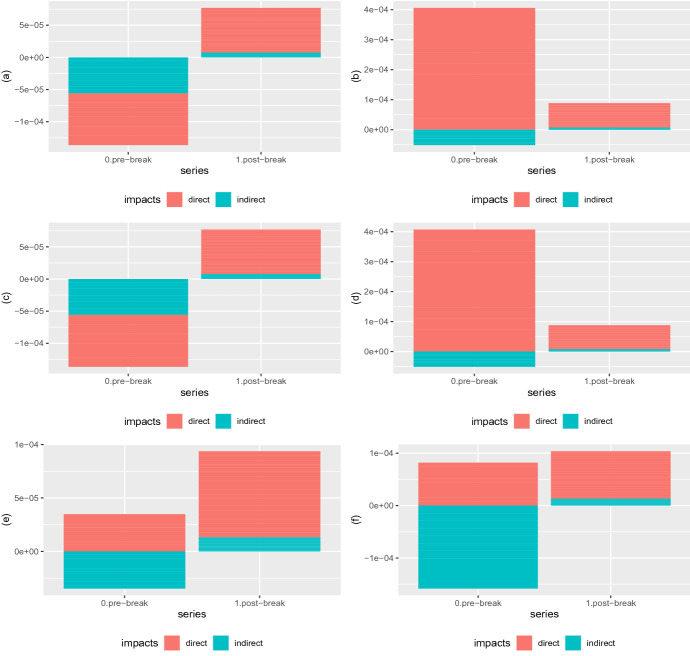
Short-term (on the left) and Long-term (on the right) Marginal Effects in terms of Log–Returns with respect to the number of deaths variable ${\mathbf {x}}_{c,t}$ for models **a**, **b** SEM2SRRE, **c**, **d** SEMSRRE, **e**, **f** SR, respectively

**Fig. 7:**
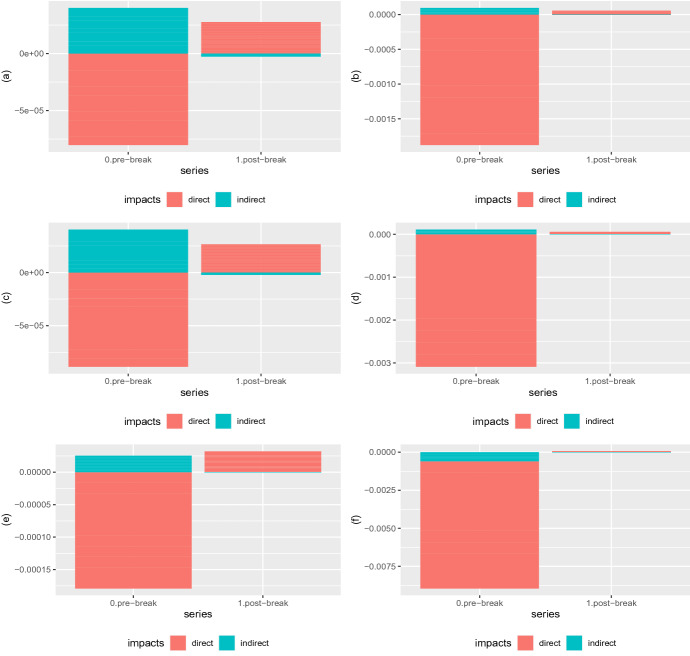
Short-term (on the left) and Long-term (on the right) Marginal Effects in terms of Volatility with respect to the number of deaths variable ${\mathbf {x}}_{c,t}$ for models **a**, **b** SEMSRRE, **c**, **d** SEMSR, **e**, **f** SR, respectively

**Fig. 8:**
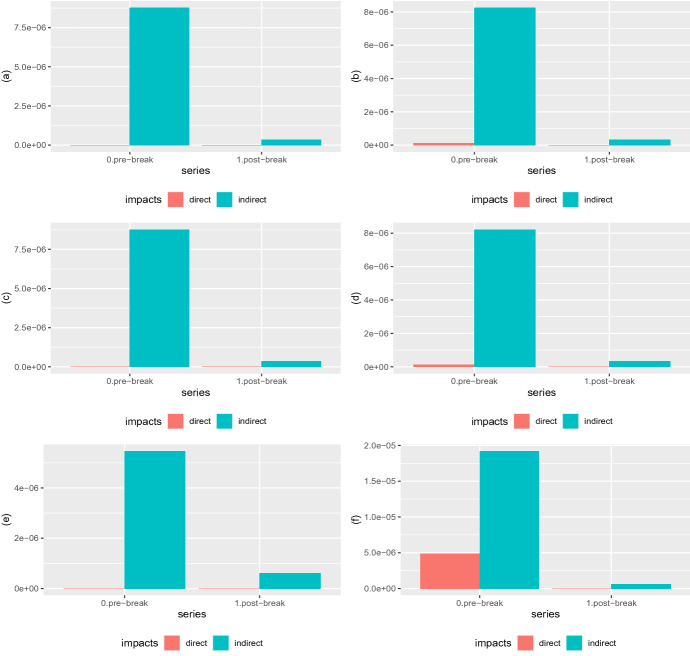
Standard deviations (SDs) of the short-term (on the left) and long-term (on the right) marginal effects in terms of log–returns for models **a**, **b** SEM2SRRE, **c**, **d** SEMSRRE, **e**, **f** SR, respectively

**Fig. 9:**
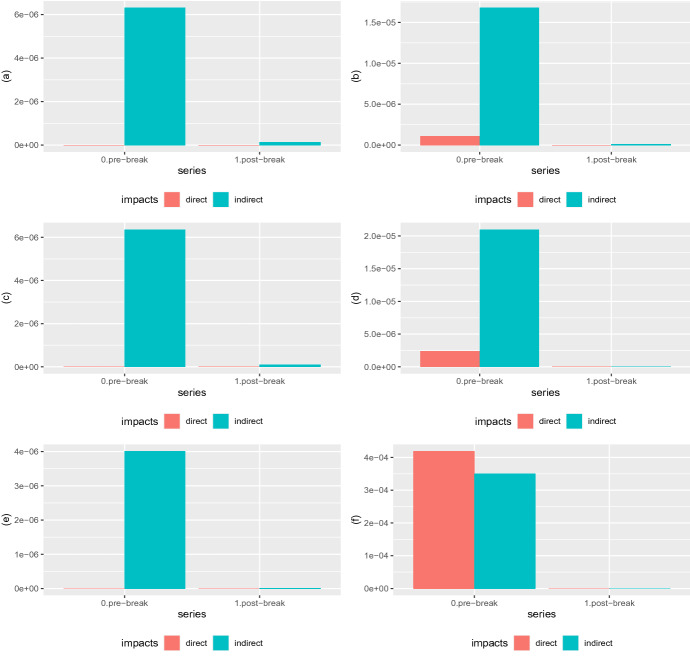
Standard deviations (SDs) of the short-term (on the left) and long-term (on the right) marginal effects in terms of volatility for models **a**, **b** SEMSRRE, **c**, **d** SEMSR, **e**, **f** SR, respectively

## Appendix A: Additional figures

See Figs. 6, 7, 8 and 9.

## Footnotes

1 We measured the decrease between mid March 2020 and the end of the month using the financial market indexes of our empirical analyses; our indexes are expressed in local currencies.

2 Again focusing on our data, about 50% of the financial markets indexes we consider have, at the end of 2020, a value larger than the one at the beginning of 2020.

3 We use data at the weekly frequency and therefore we include a one period lag in our analyses.

4 Transformed series are all stationary.

5 Similar figures for the other specifications are included in the Appendix, see Figs. 6 and 7.

6 Similar figures for the other specifications are included in the Appendix, see Figs. 8 and 9.
